# ZGDHu-1 promotes apoptosis of mantle cell lymphoma cells

**DOI:** 10.18632/oncotarget.14274

**Published:** 2016-12-27

**Authors:** Liannv Qiu, Jinlin Liu, Zhenni Wang, Sufeng Chen, Weixiao Hu, Qiang Huang, Yonglie Zhou

**Affiliations:** ^1^ Department of Clinical Laboratory, Zhejiang Provincial People's Hospital, Hangzhou Medical College, Hangzhou, Zhejiang, China; ^2^ College of Pharmaceutical Science, Zhejiang University of Technology, Hangzhou, Zhejiang, China; ^3^ Department of Hematology, Zhejiang Provincial People's Hospital, Hangzhou Medical College, Hangzhou, Zhejiang, China

**Keywords:** N, N’-di-(m-methylphenyi)-3, 6-dimethyl-1, 4-dihydro-1, 2, 4, 5-tetrazine-1, 4-dicarboamide, mantle cell lymphoma, cell cycle, apoptosis, NF-κB

## Abstract

Mantle cell lymphoma (MCL) is a well-defined aggressive Non-Hodgkin-lymphoma with short survival rates and remains incurable to date. Previously, we demonstrated the antitumor activity of ZGDHu-1(N, N’-di-(m-methylphenyi)-3, 6-dimethyl-1, 4-dihydro-1, 2, 4, 5-tetrazine-1, 4-dicarboamide) in chronic lymphocytic leukemia. In this study, ZGDHu-1 shows potent anti-lymphoma activity in MCL cells. ZGDHu-1 significantly induces cell cycle G2/M phase arrest and apoptosis in MCL cells. ZGDHu-1 reduces the protein levels of Mcl-1, Bcl-XL and cyclin D1. Importantly, ZGDHu-1 inhibits TNFα-induced IkBa phosphorylation, p65 nuclear translocation and NF-kB downstream target gene expression in MCL cells. MCL samples expressing high levels of Bcl-2 and high Bcl-2/Bax ratios tend to be less effective to ZGDHu-1. Together, these results suggest that ZGDHu-1 could inhibit the NF-kB signaling pathway partly, which may lead to the suppression of cell proliferation and the induction of apoptosis in MCL cells. Thus, our studies provide evidence of the potential of ZGDHu-1 in treating mantle cell lymphoma.

## INTRODUCTION

Mantle cell lymphoma (MCL) is a highly aggressive form of Non-Hodgkin-lymphomas (NHL) with a median survival of approximately 5 years and accounts for 6-8% of NHL [1–3]. Although some patients exhibit clinically indolent progression, MCL is generally aggressive, with most patients in stage III or IV at diagnosis [3]. Although conventional chemotherapy results in an overall 60-80% response, most patients relapse and succumb to MCL [4]. Patient responses to currently available therapies, including monoclonal antibody therapy and high-dose chemotherapy followed by stem cell transplantation, have been limited [5–8]. Thus far, no therapy has been sufficiently effective in extending the overall survival time of patients with MCL. Therefore, novel treatments that maximize therapeutic benefits and minimize toxicities are desperately needed.

MCL is characterized by chromosomal translocation (11; 14) (q13; q32). Cyclin D1 is regulated by NF-κB and regulates the G1-S transition of the cell cycle [9]. Though cyclin D1 overexpression has become the hallmark of MCL, it is not sufficient for the development of MCL. Accumulating evidence suggests that MCL is often detrimental to other cellular processes, such as the apoptosis and DNA repair. Apoptosis plays an important role in cancer development. MCL cells evade apoptosis by up-regulating the expression of anti-apoptotic proteins [9]. We know that NF-κB activation plays a critical role in MCL cell survival and leads to the overexpression of several anti-apoptotic molecules [9–13]. Among these, anti-apoptotic members of the Bcl-2 family appears to be particularly important in the the development of MCL [10]. Inhibiting NF-κB activation has been shown to induce cell-cycle arrest and cell death in MCL cells [11–13]. Therefore, the NF-κB pathway is an attractive target for therapeutic intervention in the onset and progression of MCL.

**Figure 1:**
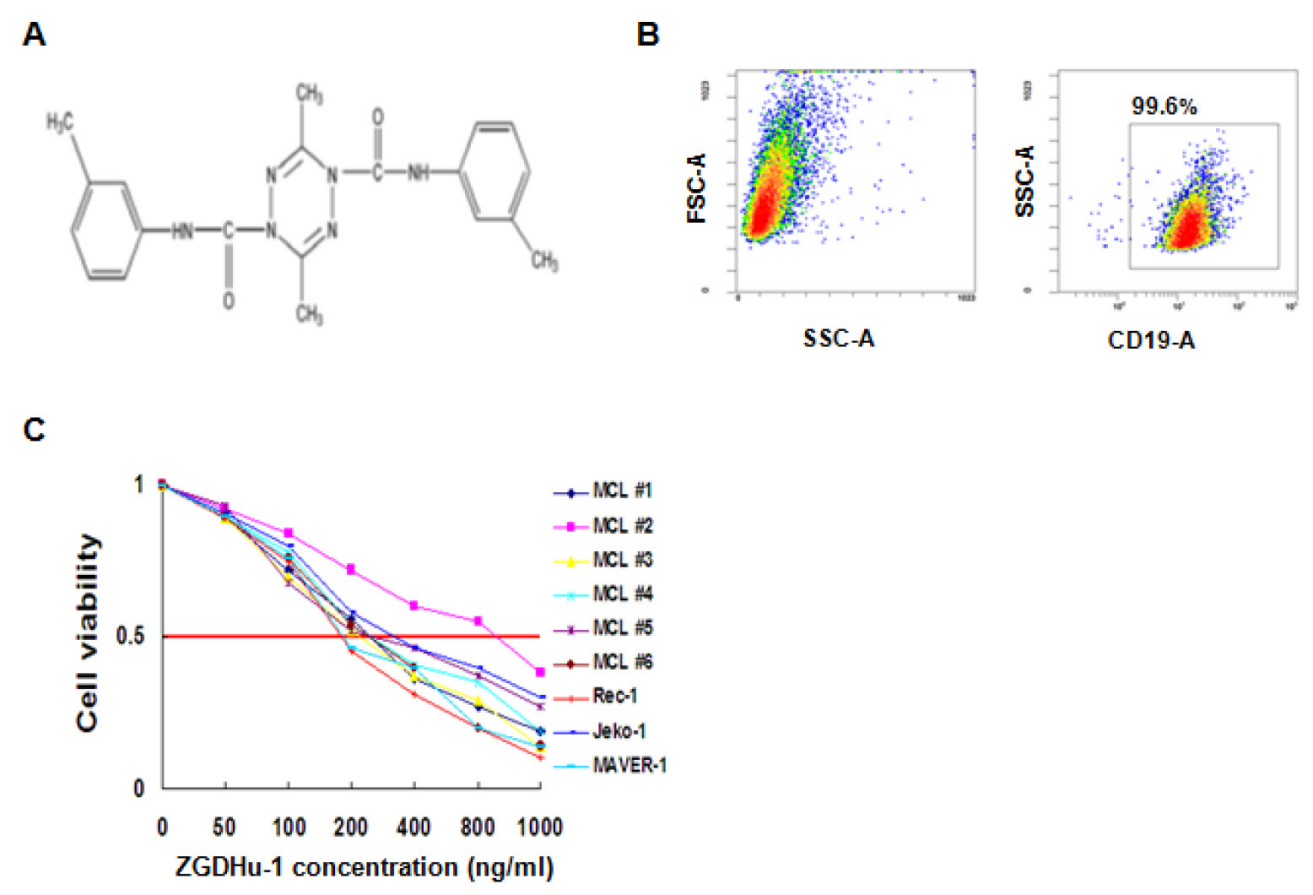
Identification of ZGDHu-1 as a potent anti-lymphoma compound in MCL cells **A**. The chemical structure of ZGDHu-1 **B**. Purity of isolated primary MCL cells **C**. Six primary MCL cells were cultured with 0 - 1000 ng/ml ZGDHu-1 for 72 h. Cell viability was measured with MTT assay.

ZGDHu-1[N, N’-di-(m-methylphenyi)-3, 6-dimethyl-1, 4-dihydro-1, 2, 4, 5-tetrazine-1, 4-dicarboamide] (Figure 1A), a tetrazine compound, synthesized by Wei-Xiao Hu (Pharmaceutical College of Zhejiang University of Technology, China) [14], possesses anti-tumor activity [15, 16] and has been identified as a potential proteasome inhibitor [17]. The effects of this novel ZGDHu-1 and its molecular mechanism(s) against MCL have not yet been investigated. We hypothesized that ZGDHu-1 exerts anti-lymphoma activity in MCL cells by inducing apoptosis via a mechanism that likely involves NF-κB regulation.

In this study, we screened 17 primary MCL patients and three different MCL cell lines to investigate the anti-lymphoma activity of ZGDHu-1. Primary MCL cells from two MCL patients (MCL #3 and MCL #12) and three MCL cell lines were further examined to study ZGDHu-1's molecular mechanism. We found that ZGDHu-1 exerted cytotoxic activity and significantly induced cell cycle G2/M phase arrest and apoptosis in MCL cells. We also demonstrated that ZGDHu-1 decreased IkBa phosphorylation and reduced the expression of Mcl-1, Bcl-XL and cyclin D1 and blocked the TNFα-induced NF-κB signaling pathway in MCL cells. These findings suggest that ZGDHu-1 induces apoptosis by inhibiting the NF-κB signaling pathway in MCL cells partly, thereby providing evidence that ZGDHu-1 is a potential therapeutic molecule that may improve MCL patients’ outcomes.

## RESULTS

### ZGDHu-1 is identified as a potent cytocoxic compound in MCL cells

To determine the sensitivity of MCL cells to ZGDHu-1, three MCL cell lines (Rec-1, Jeko-1, MAVER-1) were treated with increasing doses of ZGDHu-1 for 72 hours. As shown in Table 1, the efficacy of ZGDHu-1 was heterogeneous on different MCL cell lines. Indeed, Rec-1 (the half maximal inhibitory concentrations (IC50) 187.6 ng/ml), Jeko-1 cells (IC50 213.4 ng/ml) were found to be highly sensitiver to ZGDHu-1 than MAVER-1 cells (IC50 358.9 ng/ml) (Figure 1C). We next investigated ZGDHu-1 sensitivity in 6 primary MCL cells obtained randomly from peripheral blood of 17 patients. The same as MCL cell lines, ZGDHu-1 induced cell death in 6 all samples. Five primary cells reduced the viabilities of MCL cells (IC 50 224.9 ng/ml for MCL #1, IC 50 187.9 ng/ml for MCL #3, IC 50 289.6 ng/ml for MCL #4, IC 50 247.9 ng/ml for MCL #5, IC 50 257.1 ng/ml for MCL #6) (Figure 1C and Supplementary Figure 1A-1B). However, MCL #2 primary MCL cells were apparently insensitive to ZGDHu-1 (IC50 809.1 ng/ml). So we evaluated the effects of ZGDHu-1 (100 - 400 ng/ml, 48 h) on the viabilities of 17 primary MCL cells using PI stainning. ZGDHu-1 reduced the viabilities of primary MCL cells in a time-dependent manner (Supplementary Figure 1B) and a dose-depependent manner (Supplementary Figure 1C). Most importantly, in our previous research, this inhibitive effect of ZGDHu-1 was not observed in normal peripheral B cells which were treated with ZGDHu-1 under the same conditions [16], suggesting that the cytotoxic effects of ZGDHu-1 on MCL cells are specific. Thus, for subsequent studies, the treatment duration was set at 48 h for 100 ng/ml and 200 ng/ml ZGDHu-1.

**Table 1:** Biological characteristics and basal mRNA relative levels of anti-apoptotic factors in primary MCL cells

Primary MCL samples	Gender	Age	PB lymphocytes /μl	Previous treatment	TP53 status	Disease status	%ZGDHu-1 cytotoxicity (48 h)	mRNA relative level			
								Mcl-1	Bcl-2	Bax	Bcl-XL
#1	M	67	22230	No	wt	D	35.7	0.82	2.46	0.72	0.87
#2	M	74	76100	No	wt	D	19.2	0.78	13.28	1.07	0.42
#3	M	69	45670	No	wt	D	66.3	0.9	2.10	1.1	1.81
#4	F	81	68700	No	wt	D	24.2	1.59	8.59	0.95	1.02
#5	M	64	56210	No	wt	D	34.7	1.09	2.14	0.32	0.37
#6	M	75	123480	No	del/wt	D	31.2	0.87	5.24	0.5	0.99
#7	M	78	85790	No	wt	D	36.5	0.94	4.01	0.37	2.14
#8	F	69	67820	No	del/mut	D	42.5	1.03	3.45	1.11	0.56
#9	M	71	35620	No	wt	D	51.7	0.45	2.79	0.50	0.49
#10	F	73	112340	No	mut	D	37.5	0.56	2.41	0.49	0.15
#11	M	83	67950	No	wt	P	27.9	0.74	8.17	0.61	0.35
#12	M	79	56890	No	UPD/ c	D	65.2	0.87	2.24	0.32	2.14
#13	M	59	69840	No	wt	D	38.9	0.68	3.14	0.47	3.13
#14	F	76	65700	No	wt	D	32.1	1.02	4.69	0.57	1.87
#15	M	74	79650	No	del/ c	D	45.8	0.54	3.01	0.29	0.56
#16	F	63	84500	No	wt	D	38.7	0.89	2.15	0.87	1.65
#17	M	82	43200	No	wt	D	46.8	1.05	2.42	0.73	2.34

Abbreviations: D, diagnosis; wt, wild type; del, deletion; mut, mutation; UPD, uniparental disomy

### ZGDHu-1 induces cell cycle G2/M phase arrest in MCL cells

**Figure 2:**
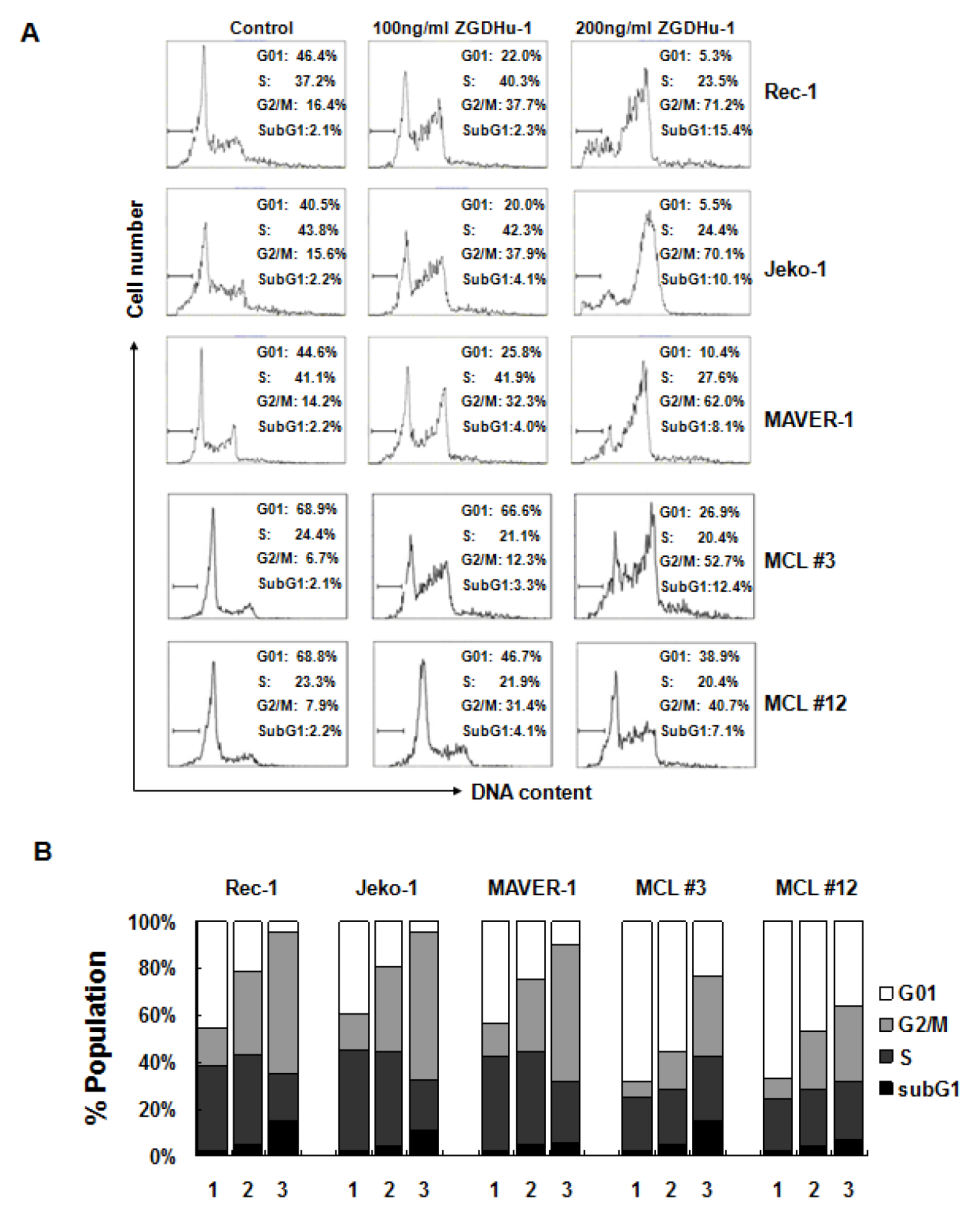
ZGDHu-1 induces cell cycle G2/M phase arrest in MCL cells **A**. MCL cells were cultured in 0.05% DMSO drug-free medium (control) or 100 - 200 ng/ml ZGDHu-1 for 48 h. The cell cycle distribution was analyzed using the Wincycle32 software. The percentage of cells in the subG1 phase is depicted in each plot. **B**. Quantifications of the proportions of cells in subG1, G01, S, and G2/M phases are listed for each experiment. ZGDHu-1 (100 - 200 ng/ml, 48 h) significantly increased the percentage of G2/M phase of MCL cells compared to DMSO controls.Values represent means ± SD for three separate experiments, each performed in triplicate.

Because ZGDHu-1 inhibits MCL cell proliferation, it is likely that ZGDHu-1 causes MCL cell cycle arrest. To test this hypothesis, we treated two random primary MCL cells (MCL #3 and MCL #12) and three MCL cell lines with ZGDHu-1 (100 - 200 ng/ml) for 48 h and measured the cell cycle distribution. Indeed, following ZGDHu-1 treatment, the percentage of cells in the G2/M phase significantly increased in a dose-dependent manner in primary MCL cells and three MCL cell lines (Figure 2A–2B and Supplementary Figure 2). The percentages of cells in the G01 and S phase were not significantly changed. We also showed that ZGDHu-1 increased the fraction of cells in the sub-G1 phase (Figure 2A–2B and Supplementary Figure 2). Together, these data suggested that ZGDHu-1 could induce cell cycle G2/M phase arrest and apoptosis in MCL cells.

### ZGDHu-1 induces apoptosis in MCL cells

**Figure 3:**
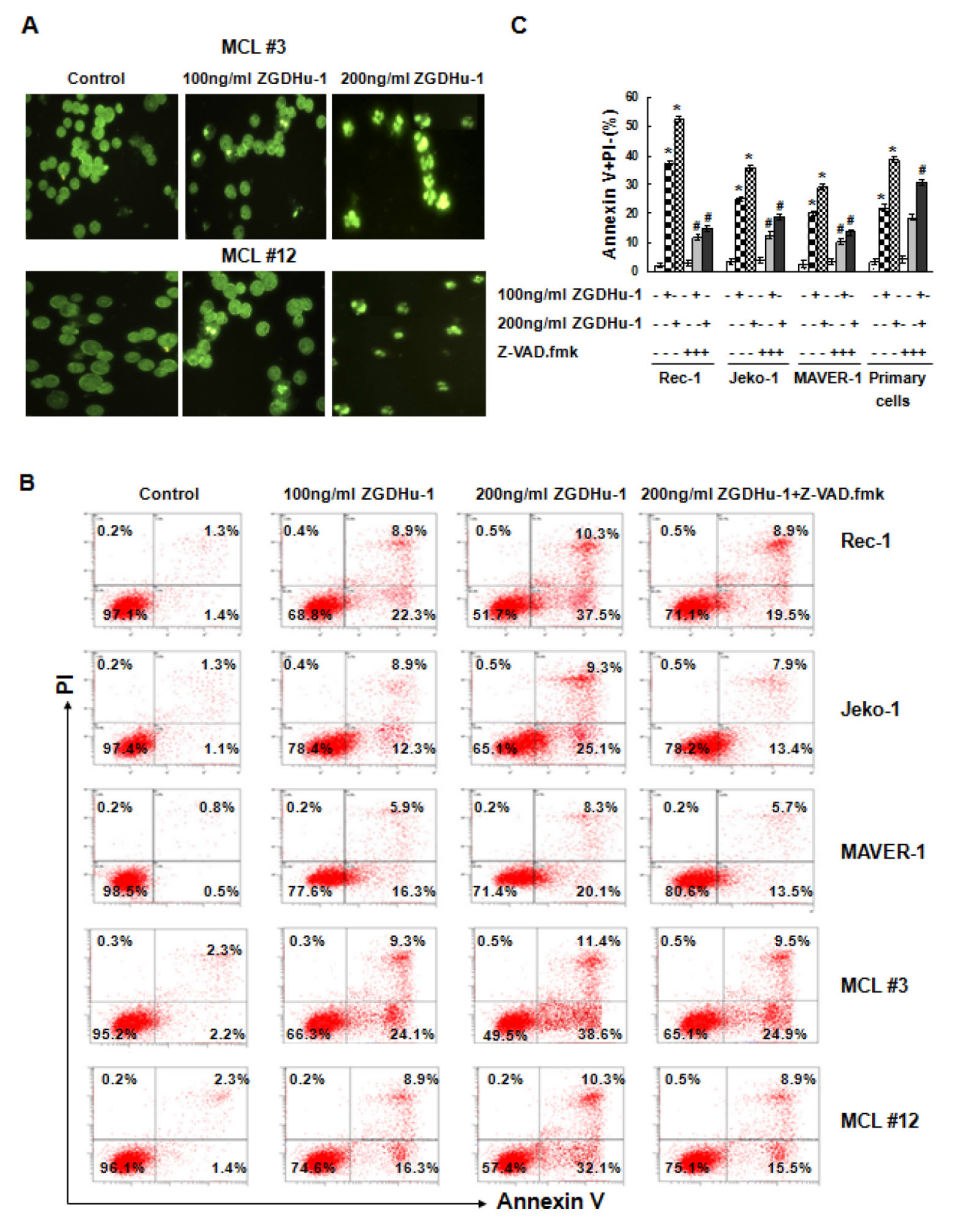

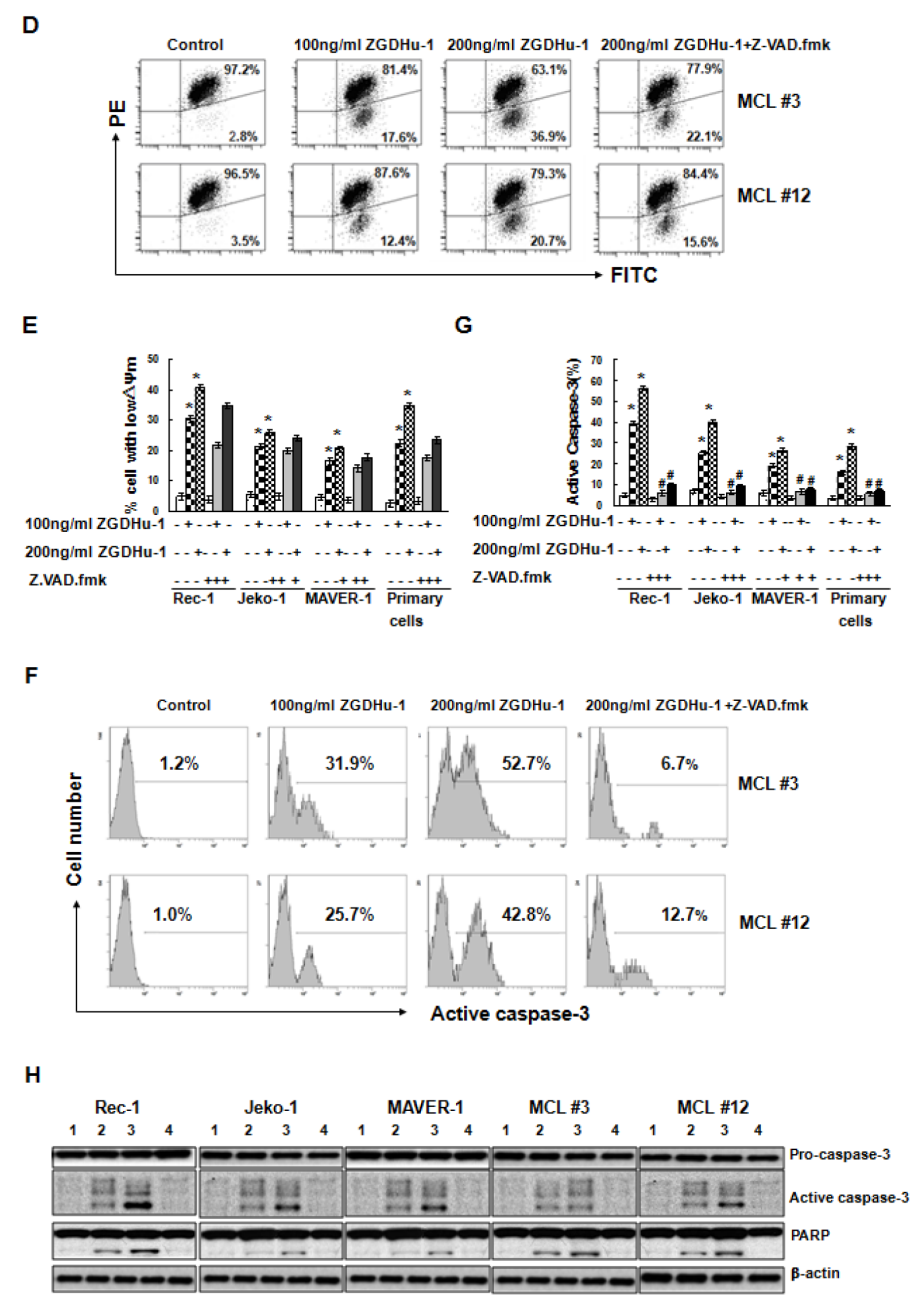
ZGDHu-1 induces apoptosis in MCL cells **A**. Apoptotic cell morphology was measured by the Hoechst33258 staining.The morphologies of primary MCL cells from two MCL patients (MCL #3 and MCL #12) changed dramatically after ZGDHu-1 (100 - 200 ng/ml) treatment for 48 h. **B**. MCL cells were cultured in 0.05% DMSO drug-free medium or 100 - 200 ng/ml ZGDHu-1 in the absence or presence of 200 mM Z-VAD.fmk for 48 h, stained with Annexin V/PI and analyzed by flow cytometry. Representative histograms of Annexin-V/PI of primary MCL cells and three MCL cell lines are displayed. **C**. Quantitative data pertaining to panel B. Percentages of Annexin V-positive cells are shown. Values represent means ± SD, each performed in triplicate. * P<0.05 between ZGDHu-1 and control; # P<0.05 between ZGDHu-1 and ZGDHu-1+Z-VAD.fmk. **D**. MCL cells were treated with ZGDHu-1 (100 - 200 ng/ml) in the absence or presence of Z-VAD.fmk for 48 h, stained with JC-1 and analyzed using flow cytometry. Representative histograms of decreased ΔΨm are shown from two MCL patients (MCL #3 and MCL #12). **E**. Quantitative data pertaining to panel D. Percentages of decreased ΔΨm values are shown. * P<0.05 between ZGDHu-1 and control; # P<0.05 between ZGDHu-1 and ZGDHu-1+Z-VAD.fmk. **F**. Active caspase-3 was analyzed by flow cytometry in MCL cells treated with ZGDHu-1 (100 - 200 ng/ml) in the absence or presence of 200 mM Z-VAD.fmk for 48 h. Representative histograms of active caspase-3 are shown from two MCL patients (MCL #3 and MCL #12). **G**. Quantitative data pertaining to panel F. Percentages of active caspase-3 are shown from two MCL patients (MCL #3 and MCL #12). * P<0.05 between ZGDHu-1 and control; # P<0.05 between ZGDHu-1 and ZGDHu-1+Z-VAD.fmk. **H**. Western blot analysis of active caspase-3 and PARP are showed in priary MCL cells and three MCL cell lines following ZGDHu-1 (100 - 200 ng/ml) treatment in the absence or presence of 200mM Z-VAD.fmk for 48 h. 1: Control; 2: 100 ng/ml ZGDHu-1; 3: 200 ng/ml ZGDHu-1; 4: 200 ng/ml ZGDHu-1+ Z-VAD.fmk.

ZGDHu-1 dramatically decreased cell viability within 48 h, suggesting that ZGDHu-1 mainly induces cell death in addition to causing cell cycle arrest. We observed that most ZGDHu-1 (100 - 200 ng/ml, 48 h) - treated MCL cells presented typical morphological changes with apoptosis, pyknosis (nucleus condensing) and karyorrhexis (nucleus fragmenting) by Hoechst 33258 staining (Figure 3A). ZGDHu-1 (100 - 200 ng/ml, 48 h) significantly induced Annexin V^+^ PI^-^ apoptotic cells in primary MCL cells and three MCL cell lines in dose-dependent manner (Figure 3B–3C and Supplementary Figure 3A). Moreover, ZGDHu-1 induced the dissipation of ΔΨm (Figure 3D–3E and Supplementary Figure 3B), the cleavage of Caspase-3 (Figure 3F–3H and Supplementary Figure 3C) and PARP (Figure 3H), which is a well-known caspase substrate, in MCL cells. It appeared that the MCL cells underwent apoptosis after ZGDHu-1 treatment.

To further confirm whether caspase-dependent apoptosis plays a prominent role in ZGDHu-1-induced apoptosis, we treated MCL cells with ZGDHu-1 in the presence of the pan-caspase inhibitor Z-VAD.fmk (200 mM) and observed that ZGDHu-1-induced Annexin V^+^ PI^-^ apoptosis, ZGDHu-1-induced caspase-3 activation as well as the cleavage of PARP was largely abrogated (Figure 3B–3C,Figure 3F–3H and Supplementary Figure 3A, 3C). But the adding of Z-VAD.fmk did not inhibit the dissipation of ΔΨm (Figure 3D–3E and Supplementary Figure 3B). Together, these results strongly suggested that ZGDHu-1 induced the caspase-dependent apoptosis of MCL cells.

### ZGDHu-1 induces changes in ROS in MCL cells

**Figure 4:**
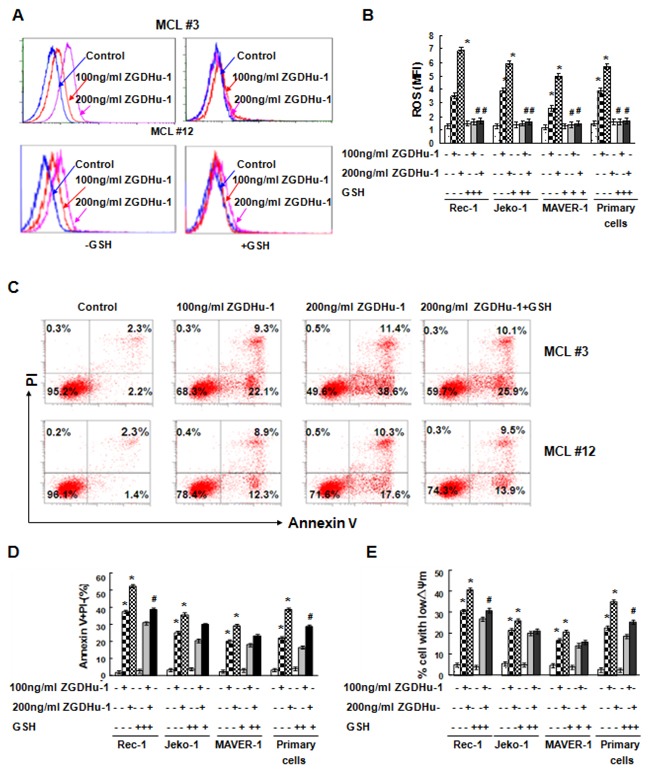
Effects of ZGDHu-1 on ROS levels in MCL cells **A**. MCL cells were cultured in 0.05% DMSO drug-free medium or 100 - 200 ng/ml ZGDHu-1 in the absence or the presence of 100 μM GSH for 48 h. DHR staining and flow cytometry were used for ROS generation. Representative ROS MFI is displayed from two MCL patients (MCL #3 and MCL #12). **B**. Quantitative data pertaining to panel A. Data revealed the effect of ZGDHu-1 on ROS MFI of primary MCL cells and three MCL cell lines in the absence or the presence of 100 μM GSH for 48 h. * *P*<0.05 between ZGDHu-1 and control; # *P*<0.05 between ZGDHu-1 and ZGDHu-1+GSH **C**. Representative histograms of Annexin-V/PI are displayed from two MCL patients (MCL 3# and MCL 12#) following exposure to ZGDHu-1 in the absence or the presence of 100 μM GSH for 48 h. **D**. Quantitative data pertaining to panel C. Data reveals the effect of GSH on ZGDHu-1-induced apoptosis in primary MCL cells and three MCL cell lines for 48 h. * *P*<0.05 between ZGDHu-1 and control; # *P*<0.05 between ZGDHu-1 and ZGDHu-1+GSH **E**. Data reveals the effect of GSH on ZGDHu-1-induced changes in the ΔΨm of MCL cells for 48 h. * *P*<0.05 between ZGDHu-1 and control. # *P*<0.05 between ZGDHu-1 and ZGDHu-1+GSH.

Because reactive oxygen species (ROS) plays a key role in apoptosis, we used DHR staining and flow cytometry to examine whether ROS levels were affected by ZGDHu-1 in MCL cells. ZGDHu-1 (100 - 200 ng/ml, 48 h) significantly induced ROS generation of MCL cells in a dose-dependent manner (Figure 4A–4B). We also tested whether the ROS scavenger glutathione (GSH) could suppress ZGDHu-1-induced apoptosis in primary MCL cells and three MCL cell lines. Pretreatment with GSH (100 μM) for 2 h could significantly block ZGDHu-1-induced ROS generation (Figure 4A–4B), and partly inhibited the pro-apoptotic effect of ZGDHu-1 on MCL cells (Figure 4C–4D) and ZGDHu-1-induced the dissipation of ΔΨm in MCL cells (Figure 4E). Taken together, these results suggested that ZGDHu-1 can promote apoptosis of MCL cells by inducing ROS generation.

### ZGDHu-1 decreases the expression levels of multiple cell cycle and apoptosis regulators in MCL cells

**Figure 5:**
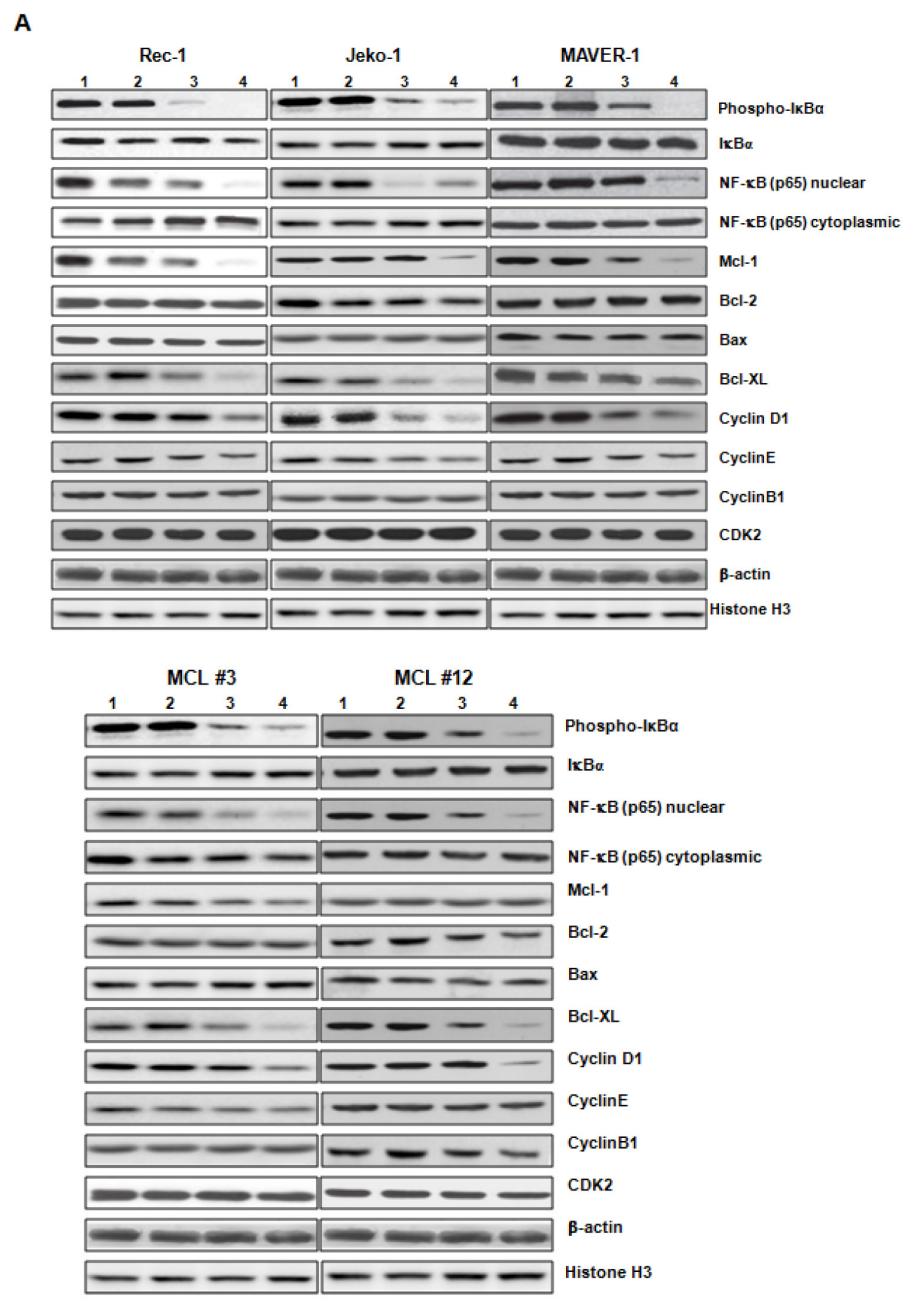

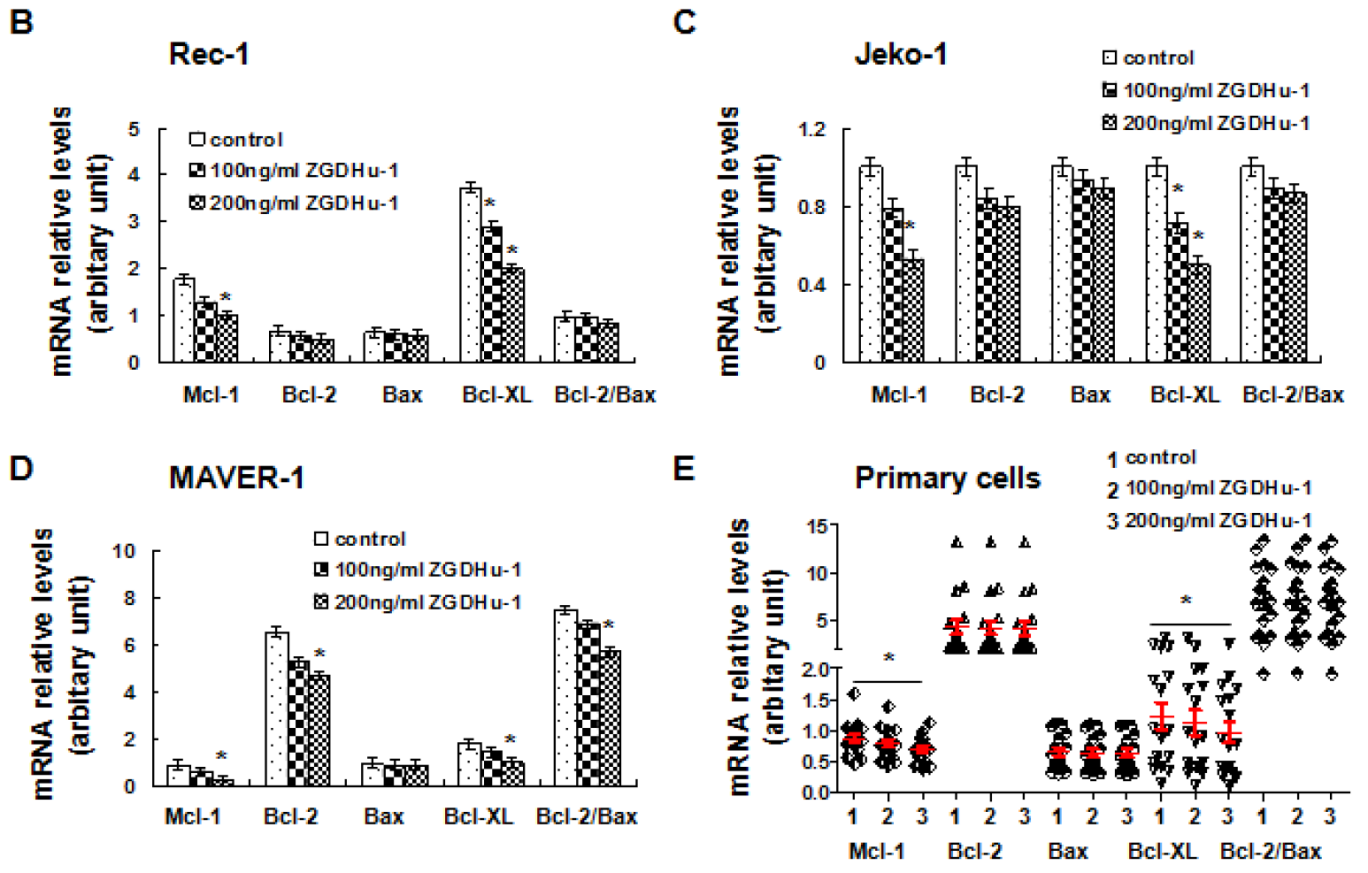
ZGDHu-1 decreases the protein levels of multiple cell cycle and apoptosis regulators in MCL cells **A**. Primary MCL cells and three MCL cell lines were cultured in 0.05% DMSO drug-free medium or 50- 200 ng/ml ZGDHu-1 for 48 h. Cells were collected and whole cell lysate or cytoplasmic/nuclear fraction was prepared for western blot analysis to detect the levels of IkBa phosphorylation, nuclear and cytoplasmic NF-κB (p65) cleaved Caspase-3, and PARP, Bcl-2, Bax, Mcl-1, Bcl-XL, cyclin B1, cyclin D1, cyclin E and CDK2. β-actin and Histone H3 were used as cytoplasmic and nuclear protein controls. 1: Control; 2: 50 ng/ml ZGDHu-1; 3: 100 ng/ml ZGDHu-1; 4: 200 ng/ml ZGDHu-1. **B**. Rec-1 cells were cultured in 0.05% DMSO drug-free medium or 100 - 200 ng/ml ZGDHu-1 for 48 h. The relative mRNA levels of Mcl-1, Bcl-2, Bax and Bcl-XL were measured by qRT-PCR following ZGDHu-1treatment in Rec-1 cells for 48 h. *P<0.05 between ZGDHu-1 and control. **C**. The relative mRNA levels of Mcl-1, Bcl-2, Bax and Bcl-XL were measured by qRT-PCR following ZGDHu-1treatment in Jeko-1 cells for 48 h. *P<0.05 between ZGDHu-1 and control. **D**. The relative mRNA levels of Mcl-1, Bcl-2, Bax and Bcl-XL were measured by qRT-PCR following ZGDHu-1treatment in MAVER-1 cells for 48 h. *P<0.05 between ZGDHu-1 and control. **E**. The relative mRNA levels of Mcl-1, Bcl-2, Bax and Bcl-XL were measured by qRT-PCR following ZGDHu-1treatment in primary MCL cells for 48 h. *P<0.05 between ZGDHu-1 and control.

Because ZGDHu-1 induced cell cycle G2/M phase arrest and apoptosis, we examined the protein levels of several cell cycle and apoptosis regulators to investigate the apoptosis mechanism triggered by ZGDHu-1 in MCL cells. In primary MCL cells and three MCL cell lines, ZGDHu-1 (100 - 200 ng/ml, 48 h) decreased the protein levels of cyclin D1, but not cyclin B1, cyclin E and CDK2, in dose-dependent manners (Figure 5A). Likewise, ZGDHu-1 decreased the protein levels of Mcl-1 and Bcl-XL, but not Bcl-2 and Bax, in dose-dependent manners in MCL #3 primary MCL cells and three MCL cell lines (Figure 5A). ZGDHu-1 decreased the Bcl-XL protein levels in dose-dependent manners in MCL #12 primary MCL cells (Figure 5A).

To confirm that the Mcl-1 and Bcl-XL protein loss were due to decrease in the transcription level, Mcl-1 and Bcl-XL mRNA levels following ZGDHu-1 treatment were measured using qRT-PCR and a-tubulin as a control. ZGDHu-1 (100 - 200 ng/ml, 48 h) caused a rapid reduction of Mcl-1 mRNA and Bcl-XL mRNA in Rec-1 (Figure 5B), Jeko-1 (Figure 5C), MAVER-1 (Figure 5D) and 17 primary cells (Figure 5E). These results confirmed that the losses of Mcl-1 and Bcl-XL protein were due to decrease on the transcription level.

### ZGDHu-1 blocks TNFα-induced NF-κB activation and the expression of anti-apoptotic proteins

It is well known that the NF-κB signaling pathway inhibits apoptosis by inducing the expression of anti-apoptotic proteins Mcl-1, Bcl-2 and Bcl-XL. NF-κB is constitutively active and levels of nuclear p65 and IkBa phosphorylation increase in MCL cells compared to normal B cells [19–20]. Traditionally, NF-κB is composed of two subunits and is normally sequestered in the cytoplasm by its inhibitor protein, IκB [21]. NF-κB activation can induce IκB phosphorylation in NF-κB exposed cells, targeting them for rapid degradation via a proteasome pathway, which releases NF-κB to the nucleus, where it binds to specific sequences in the promoter regions of genes [22]. To investigate the mechanism by which ZGDHu-1 inhibits the expression of anti-apoptotic proteins, we tested whether ZGDHu-1 inhibits the NF-κB signaling pathway. Initially, we examined the effect of ZGDHu-1 on IkBa phosphorylation, a major target molecule of the NF-κB signaling pathway. The results indicated significant dose-dependent down-regulation of IkBα phosphorylation and nuclear p65 following ZGDHu-1 treatment in primary MCL cells and three MCL cell lines (Figure 5A). But ZGDHu-1 did not change the protein levels of Bcl-2, Bax and cytoplasmic p65 of primary MCL cells and three MCL cell lines (Figure 5A).

**Figure 6:**
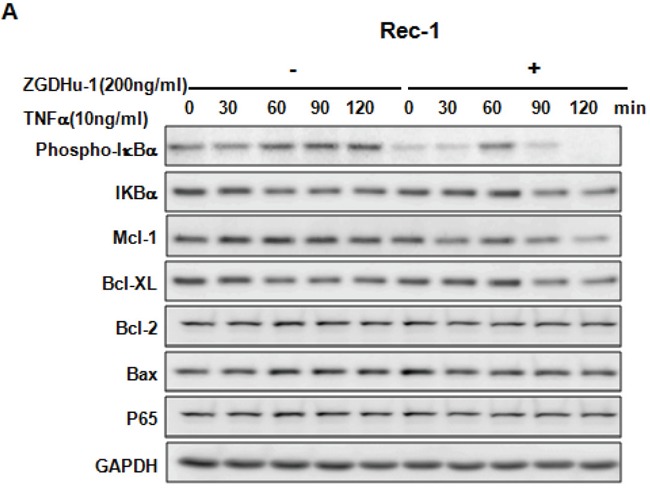

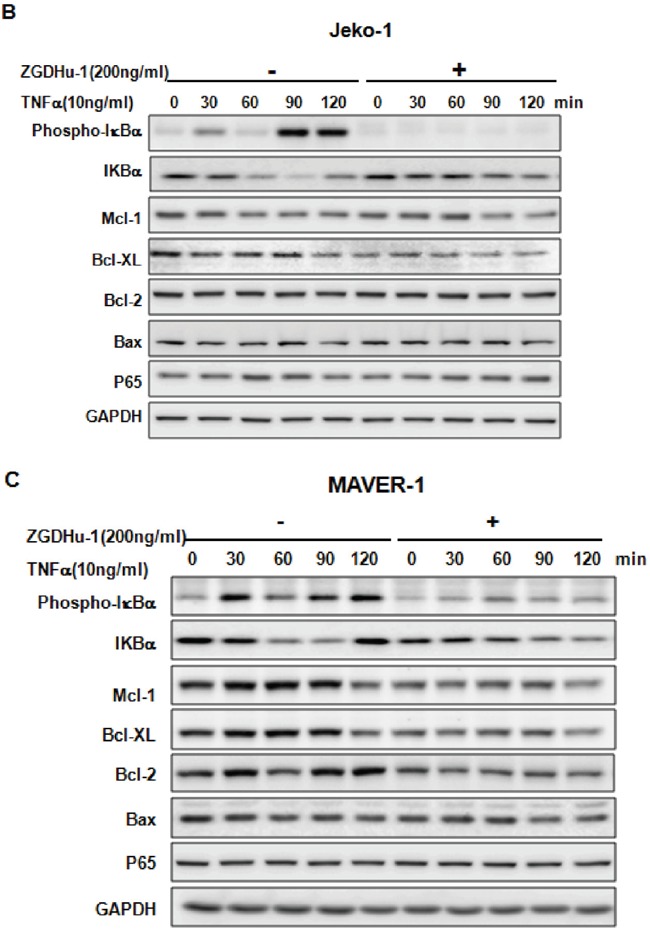

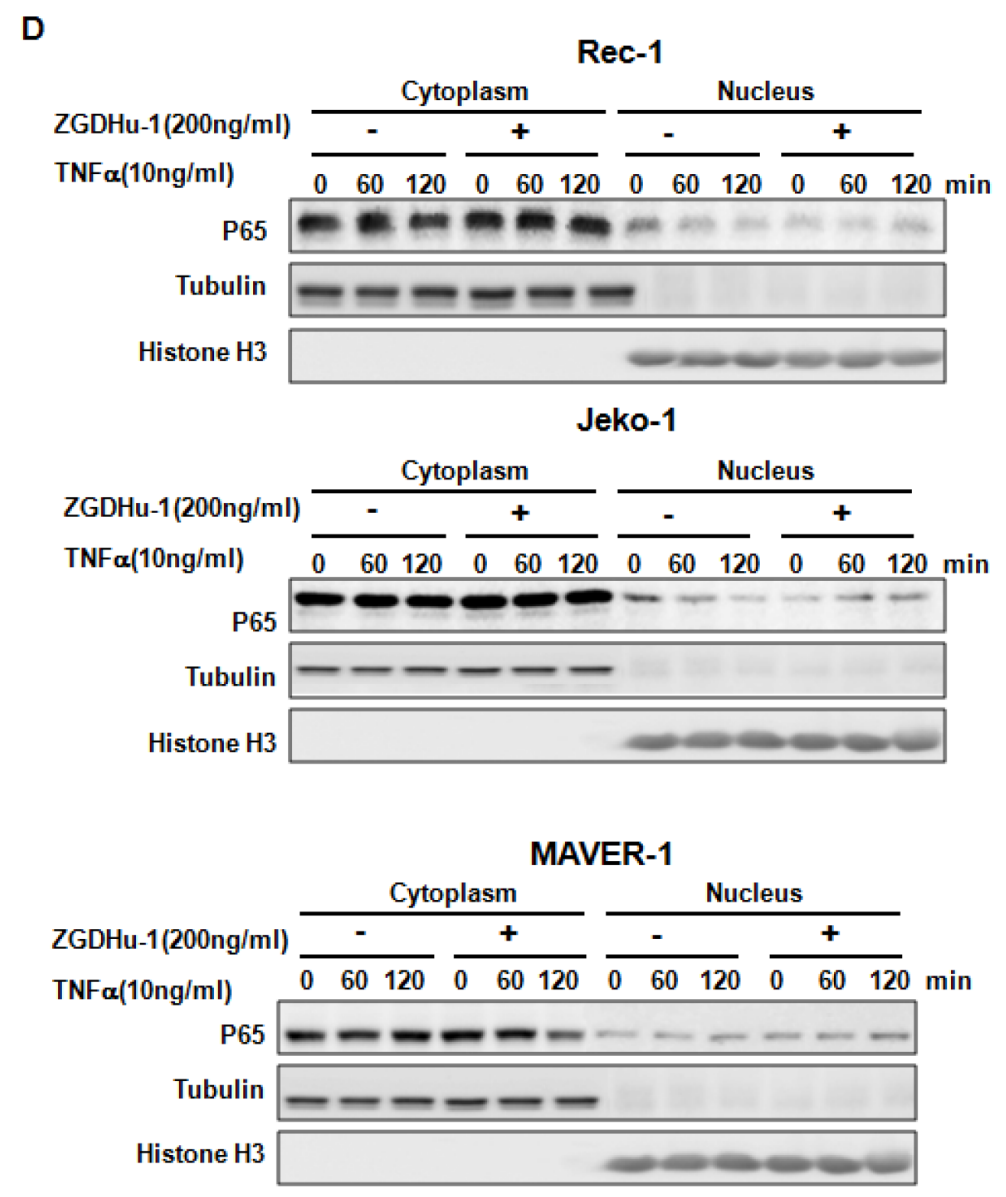
ZGDHu-1 blocks the TNFα-induced NF-κB signaling pathway and anti-apoptosis protein expression in three MCL cell lines **A**. REC-1 cells were first treated with or without ZGDHu-1 (200 ng/ml) for 24 h. Following that, TNFα (10 ng/ml) was added for 30 to 120 min. The cell lysates were collected for western blot analysis to detect the protein levels of phospho-IkBa, IkBa, Mcl-1, Bcl-XL, Bcl-2, Bax, and p65. GAPDH was used as the loading control. **B**. The protein levels of phospho-IkBa, IkBa, Mcl-1, Bcl-XL, Bcl-2, Bax, nuclear and cytoplasmic p65 in Jeko-1 cells were deteced as stated above. **C**. The protein levels of phospho-IkBa, IkBa, Mcl-1, Bcl-XL, Bcl-2, Bax, nuclear and cytoplasmic p65 in MAVER-1 cells were deteced as stated above. **D**. Three MCL cell liness were first treated with or without ZGDHu-1 (200 ng/ml) for 24 h. Then, TNFα (10 ng/ml) was added for 60 and 120 min. The nuclear and cytoplasmic fractions were collected for p65 detection. Tubulin and Histone H3 were used as cytoplasmic and nuclear protein controls.

We further evaluated whether ZGDHu-1 inhibited TNFα-induced p65 nuclear translocation in three MCL cell lines. As expected, TNFα (10 ng/ml) activated the NF-κB signaling pathway in three MCL cell lines, as indicated by the phosphorylation of IkBa, the degradation of IkBa and the induction of Mcl-1 and Bcl-XL in Rec-1 (Figure 6A), Jeko-1 (Figure 6B) and MAVER-1 (Figure 6C). ZGDHu-1 (200 ng/ml, 24 h) almost completely blocked the phosphorylation of IkBa, the degradation of IkBa and the induction of Mcl-1 and Bcl-XL proteins in Rec-1 (Figure 6A), Jeko-1 (Figure 6B). ZGDHu-1 did not affect the protein levels of Bcl-2, Bax and p65 in three MCL cell lines (Figure 6A–6C).

P65 nuclear translocation is a symbol for NF-κB activation [21]. We further used western blot analysis to evaluate whether ZGDHu-1 inhibits TNFα-induced p65 nuclear translocation in MCL cells. As indicated in Figure 6D, ZGDHu-1 significantly inhibited TNFα-induced p65 nuclear translocation at 60 to 120 min in three MCL cell lines. Overall, these results suggest that ZGDHu-1 suppresses cell growth and induces apoptosis by inhibiting the NF-κB signaling pathway in MCL cells.

### Bcl-2 expression confers resistance to ZGDHu-1

**Figure 7:**
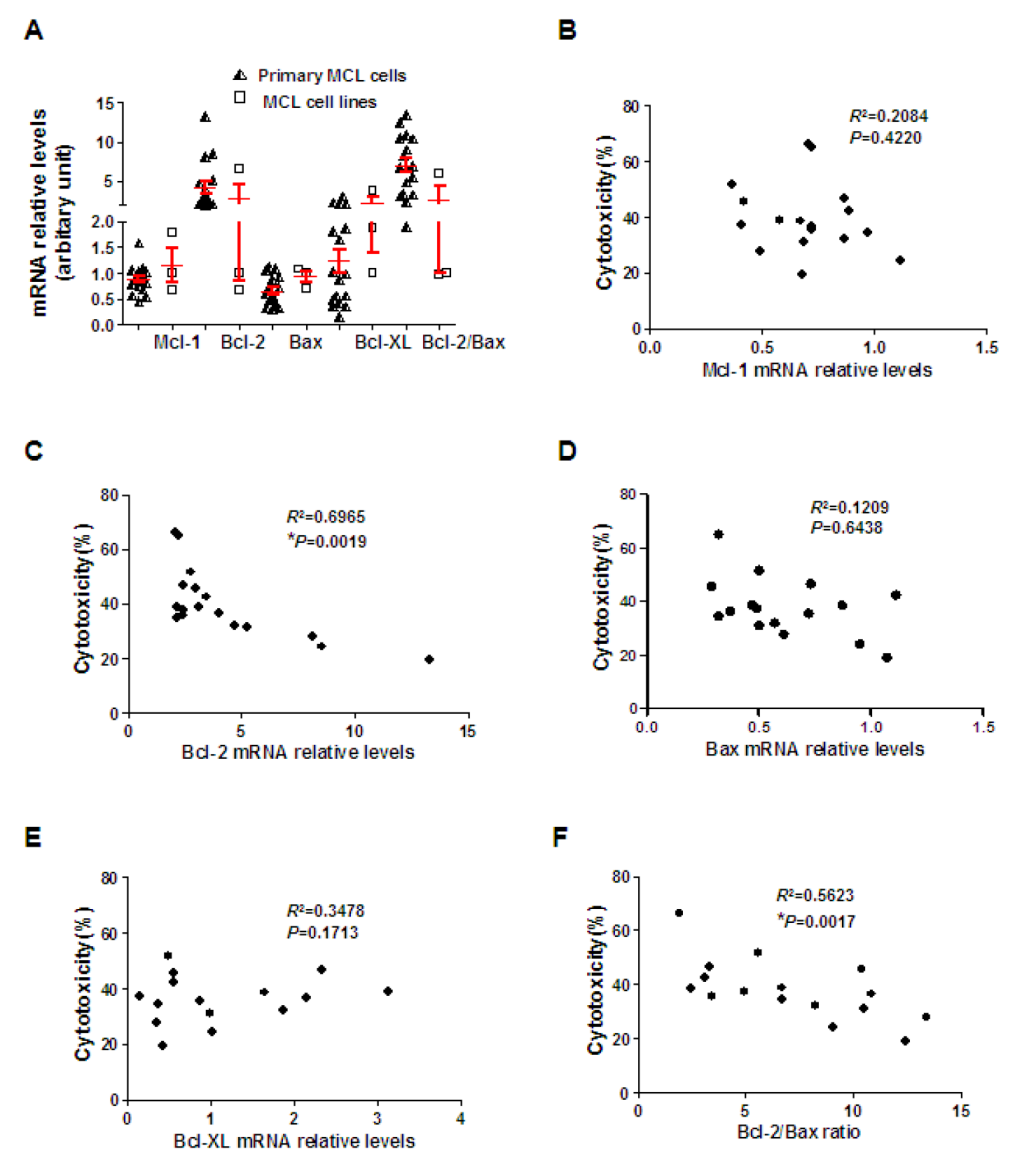
Bcl-2 expression inversely correlates with ZGDHu-1 sensitivity **A**. Mcl-1, Bcl-2, Bax and Bcl-XL mRNA relative levels in primary MCL and three MCL cell lines were detected by qRT-PCR using β-actin as a loading control. **B**. Correlation between Mcl-1 mRNA relative levels and ZGDHu-1 cytotoxicity in primary MCL cells. **C**. Correlation between Bcl-2 mRNA relative levels and ZGDHu-1 cytotoxicity in primary MCL cells. **D**. Correlation between Bax mRNA relative levels and ZGDHu-1 cytotoxicity in primary MCL cells. **E**. Correlation between Bcl-XL mRNA relative levels and ZGDHu-1 cytotoxicity in primary MCL cells. **F**. Correlation between Bcl-2/Bax ratio and ZGDHu-1 cytotoxicity in primary MCL cells.

We know Mcl-1, Bcl-XL, Bcl-2 and Bax play controlling roles in the survival of MCL cells [23]. Our results indicated that ZGDHu-1 reduced Mcl-1 and Bcl-XL protein levels in primary MCL cells. Next, we evaluated putative differences in the basal mRNA relative levels of these proteins in correlation with sensitivity to ZGDHu-1 in primary MCL cells (Table 1, Figure 7A). Interestingly, we observed that Bcl-2 mRNA levels and Bcl-2/Bax ratios were inversely correlated with ZGDHu-1 sensitivity (Figure 7C, 7F), thus indicating ZGDHu-1 less effective in Bcl-2*^high^* primary MCL cells. However, no significant association was observed between Mcl-1 mRNA levels (Figure 7B), Bax mRNA levels (Figure 7D), Bcl-XL mRNA levels (Figure 7E) and ZGDHu-1 sensitivity.

As we observed that high levels of Bcl-2 conferred less effective to ZGDHu-1, we postulated whether ZGDHu-1 could less effective in Bcl-2*^high^* MCL cell lines. To prove our surmise, we treated the representative Bcl-2*^high^* cell line MAVER-1 and Bcl-2*^low^* cell line REC-1 with ZGDHu-1 (Table 2). As expected, the results indicated Bcl-2*^low^* cell line REC-1 was sensitizer to ZGDHu-1 than Bcl-2*^high^* cell line MAVER-1 (Figure 1C and Figure 3C, 3E, 3G).

**Table 2:** Basal mRNA relative levels of anti-apoptotic factors in three MCL cell lines

MCL cell lines	%ZGDHu-1 cytotoxicity (48 h)	mRNA relative level			
		Mcl-1	Bcl-2	Bax	Bcl-XL
Rec-1	35.7	1.79	0.72	0.7	0.89
Jeko-1	52.2	1.0	1.0	1.0	1.0
MAVER-1	55.3	0.68	6.55	1.08	3.77

## DISCUSSION

In this study, we found that ZGDHu-1 showed great cytotoxicity in 17 primary MCL cells and three different MCL cell lines and identified ZGDHu-1 as a potent anti-lymphoma compound to MCL. Some differences in sensitivity to ZGDHu-1 were observed among primary MCL cells, but not in B cells from healthy donors [16]. ZGDHu-1 inhibited MCL cell proliferation by decreasing the protein levels of cyclin D1 and inducing cell cycle G2/M phase arrest. More importantly, ZGDHu-1 induced apoptosis of MCL cells by decreasing the anti-apoptotic protein levels of Mcl-1 and Bcl-XL. We demonstrated that ZGDHu-1 inhibited the activation of the TNFα-induced NF-κB signaling pathway and the induction of these anti-apoptotic proteins.

The intracellular redox status, which depends on both the GSH levels and ROS generation, is important in stabilizing mitochondrial functions. The mitochondrial dysfunction associated with ROS induced the activation of pro-apoptotic proteins, then led to caspase-dependent or caspase-independent apoptosis [24]. ZGDHu-1 significantly induced ROS generation of MCL cells and GSH could suppress ZGDHu-1-induced apoptosis in MCL cells. These results suggested ZGDHu-1 could regulate mitochondria by elevating the ROS levels and affecting the intracellular redox status. Furthermore, our data also showed that ZGDHu-1 decrease the ΔΨm, induced the cleavage of caspase-3 and PARP in MCL cells. Taken together, these findings showed that ZGDHu-1 ultimately activated the mitochondrial apoptotic pathway and caused caspase-dependent apoptosis in MCL cells, which consistent with previous reports [16, 25].

Cyclins and the CDK family play important roles in cell-cycle regulation and cell replication. Coupled with CDK, cyclin D1 regulates the G1-S transition of the cell cycle and is involved in the regulation of MCL cell proliferation. In this study, our results show that ZGDHu-1 significantly decreased cyclin D1 protein levels and induced cell cycle G2/M phase arrest in MCL cells. The suppression of cyclin D1 by ZGDHu-1 resulted in the suppression of MCL cell proliferation. Because NF-κB is well known to mediate antiapoptotic effects, elicits cell-cycle arrest and contributes to the stabilities of cyclin D1 in MCL cells [9], we examined whether suppression of NF-κB by ZGDHu-1 could lead to apoptosis.

To further elucidate the mechanism of ZGDHu-1-induced apoptosis in MCL cells, we investigated the important apoptotic regulators which are known to be regulated by NF-κB [26–27]. NF-κB activity is commonly elevated in CLL [28], MCL [9, 29], and other solid tumors [30]. The activation of the NF-κB signaling pathway is appreciated as a key mechanism for cell proliferation [31–32] and apoptosis [33–34]. We demonstrated that ZGDHu-1 inhibited the TNFα-induced expression of Bcl-XL and Mcl-1, IκBa phosphorylation and nuclear p65 levels. In the absence of exogenous TNFα, ZGDHu-1 in MCL cells also inhibited IκBa phosphorylation and nuclear p65 levels and ultimately suppressed the expression of proliferation (cyclin D1) and survival (Bcl-xL, Mcl-1) molecules. These findings suggested that ZGDHu-1 induced apoptosis most likely through the inhibition of NF-κB signaling pathway in MCL cells.

Our results indicated that ZGDHu-1 induced cell cycle G2/M phase arrest and caused caspase-dependent apoptosis in MCL cells, in consistent with similar experiments performed with CLL cells [16]. In CLL, ZGDHu-1-induced apoptosis is promoted by anti-apoptotic Bcl-2. Herein, we noticed that Bcl-2 was highly constitutively expressed in MCL patients, in accordance with other studies [35]. However, we did not observe Bcl-2 down-regulation in primary MCL cells following ZGDHu-1 treatment, suggesting a distinct mechanism for apoptosis induction in MCL cells. In this study, Mcl-1 and Bcl-XL reductions caused by ZGDHu-1 may account for the observed apoptosis. Nevertheless, although similar reductions in Mcl-1 mRNA levels were observed among all primary MCL cases, a variable degree of cytotoxic response to ZGDHu-1 could be found, suggesting that other mechanisms may be involved in ZGDHu-1-induced apoptosis of MCL cells. Remarkably, we found that high Bcl-2 expression levels and Bcl-2/Bax ratios correlated to reduced responses to ZGDHu-1 in primary MCL cells. Furthermore, Next we continue to study whether the targeting Bcl-2 can improve the sensitivity of ZGDHu-1 in Bcl-2*^high^* MCL cells.

In conclusion, this is the first report evaluating the effects of a novel tetrazine compound ZGDHu-1 on MCL. Our results show that ZGDHu-1 can potently inhibit cell proliferation and induce apoptosis in MCL cells through the inhibition of NF-κB regulated anti-apoptotic genes expression *in vitro*. In addition, results show the anti-lymphoma activity of ZGDHu-1 in MCL cells was on the targeting NF-κB pathway. Our research thus provides evidence and rationale regarding the potentially therapeutic effects of ZGDHu-1 and the possibility that treatment with this molecule may improve the outcomes of MCL patients.

## MATERIALS AND METHODS

### Patients

Seventeen MCL patients (12 males and 5 females) aged 59-83 years (with a median age of 73 years) were enrolled in this study. The biological characteristics of these cases are shown in Table 1. Patients with MCL were identified on the basis of morphologic, immunophenotypic, and molecular criteria according to World Health Organization (WHO) lymphoma classification. Only those patients who had not received previous treatments within the last 6 months were included in the study. All 17 patients were collected prior to the commencement of any treatment. Age-matched controls were obtained from 10 healthy donors. Ethical approval for this project, including informed consent from patients, was granted based on the guidelines of the Zhejiang Provincial People's Hospital research ethics committee.

### Cell lines and cell culture

Three human MCL cell lines (MAVER-1, Jeko-1 and Rec-1) were obtained from the American Type Culture Collection (ATCC) (Manassas, VA). Jeko-1 cells were cultured in RPMI 1640 medium supplemented with 20% FBS, 20 U/ml penicillin, and 20 U/ml streptomycin. MAVER-1 and Rec-1 cells were cultured in RPMI 1640 medium supplemented with 10% FBS, 20 U/ml penicillin, and 20 U/ml streptomycin. All cells were maintained at 37 °C with 5% CO_2_ in a humidified atmosphere.

### Reagents and instruments

The ZGDHu-1 compound (purity >95%) was kindly provided by Dr Wei-Xiao Hu. ZGDHu-1 was dissolved in a 1 mg/ml stock solution of dimethyl sulfoxide (DMSO) and stored at -20°C. Antibodies (used in western blot analysis) against Bcl-2, Bcl-XL, Bax, Mcl-1, cyclin D1, cyclin B1, cyclin E, cyclin-dependent kinase2 (CDK2), NF-κB (p65), caspase-3, cleaved Caspase-3, poly ADP-ribose polymerase (PARP ), IκBα and β-actin were purchased from Cell Signaling Biotechnology (Beverly, MA, USA), The anti-histone H3 antibody was purchased from Abcam (Abcam, Cambridge, UK), and PerCP CY 5.5-conjugated anti-human CD19 (ID3), phycoerythrin (PE)-conjugated anti-active caspase-3 (C92-605) and PE mouse immunoglobulin G1k (IgG1 k) isotopes control were obtained from American Beckman-Coulter Inc. DMSO, 3-(4,5-dimethylthiazol-2-yl)-2,5-diphenyl tetrazolium bromide (MTT), dihydrorhodamine-123 (DHR), broad spectrum caspase inhibitor benzyloxycarbonyl – Val – Ala – Asp - fluoromethylketone (Z-VAD.fmk) and Ficoll-Hypaque were all purchased from Sigma Aldrich Inc. (St. Louis, MO, USA). The rhodamine-conjugated monoclonal active caspase-3 antibody was from BD Pharmingen (USA). The annexin V and propidium iodide (PI) apoptosis assay kit, DNA Prep™ reagent Kit and the IntraPrept™ permeabilization kit were from Beckman Coulter (USA) and the Immunotech Company (France), respectively. JC-1 (5,5’,6,6’-tetrachloro-1,1’,3,3’-tetrethyl benzimidalyl carbocyanine iodide) was from BioTeam Inc. Flow cytometry was performed on a Beckman Coulter NAVIOS FACS (Miami, FL, USA).

### Lymphocyte purification and culture

Primary MCL cells were obtained from peripheral blood samples of MCL patients who were diagnosed according to the WHO criteria. Lymphocytes were isolated using Ficoll-Hypaque gradient centrifugation according to the manufacturer's instructions. After 1 h of incubation at 37°C in 5% CO_2_, adhesive mononuclear cells were removed. T lymphocytes were removed using anti-CD3 dynabeads (Dynal, Merseyside, UK). Purification of B lymphocytes was assessed by flow cytometry with anti-CD19 antibodies. This cell preparation contained approximately 95% CD19 positive cells (Figure 1B).

### Hoechst 33258 staining

For Hoechst 33258 staining, MCL cells were seeded into 6-well plates at 5×10^5^ cells per well. After ZGDHu-1 treatment at the indicated times, the cells were washed with serum-free RPMI 1640 and subsequently with 1× phosphate-buffered saline (PBS; DingGuo Biotechnology Co., Ltd., Beijing, China), after which they were fixed with fixative (methanol:glacial acetic acid 3:1) for 5 min at 4°C and stained with 10 μg/ml of Hoechst 33258 (Applygen Tech Inc, Beijing, China) for 10 min. Morphological changes associated with apoptosis was observed using fluorescence microscopy (Nikon Y-THS, Japan) with 350- to 370-nm excitation wavelengths and emission detection at 465 nm.

### Cell viability assay

Cell viability was determined as described using PI stainning and flow cytometer [18]. The effects of ZGDHu-1 on the viability of MCL cells were assayed using MTT assay. The optical density was measured using a microplate reader M680 (Bio-Rad, Hercules, CA, USA) and reference and test wavelengths of 630 nm and 570 nm, respectively. All experiments were performed in triplicate and repeated at least three times. The cell viability was expressed as a percentage of the DMSO-treated control samples.

### FACS

Primary MCL cells were stained with anti-CD19-PerCP CY 5.5 and anti-active caspase-3-PE. The stained cells were analyzed using NAVIOS FACS. Then, 10,000 cells were counted for each sample and CD19^+^ cell population was gated for analysis as follows. The effects of ZGDHu-1 on apoptosis in MCL cells were evaluated via annexin V/PI assay, mitochondrial membrane potentials (ΔΨm), active caspase-3 and cell cycle analysis. The annexin V/PI assay was performed according to the manufacturer's instructions, and only annexin V-positive (+) and PI-negative (−) cells were defined as apoptotic. ΔΨm was measured using JC-1 dye. The intracellular accumulation of ROS was assessed using DHR fluorescent dye. The percentage of active caspase-3-positive cells was calculated using NAVIOS FACS.

### Cell cycle analysis

A DNA Prep™ reagent Kit was used to evaluate alterations in the cell cycles of MCL cells. After the designated treatments, MCL cells were harvested and washed with PBS solution. 500 μl of DNA Prep LPR was added, and after 8 s, 500 μl of DNA Prep stain was added and then remained for 15 min at room temperature. Finally, the cells were analyzed by NAVIOS FACS, and the fractions of the cell population in the G1/G0 (G01), S, and G2/M phases were quantified via Wincycle 32 software. The sub-G1 fraction was determined from the total event count.

### Protein extraction and western blot analysis

Following incubation with different concentrations ZGDHu-1, MCL cells were lysed, and proteins were extracted and quantitated using a bicinchoninic protein assay kit (DingGuo Biotechnology Co., Ltd.). The supernatant constituted the cytoplasmic protein fraction. The pellet was washed once with Cytoplasm Lysis Buffer and extracted using Nuclear Lysis Buffer (50 mM Tris-HCl pH 8.1, 10 mM EDTA, 1% SDS, 1% P-8340 (Sigma, St. Louis, MO)). The proteins were loaded into wells of an 8 or 12% SDS-PAGE, electrophoresed and transferred onto a nitrocellulose membrane (DingGuo Biotechnology Co., Ltd.). The membrane was incubated with the appropriate primary antibody and then washed and incubated with horseradish peroxidase-conjugated secondary antibody (Cell Signaling Technology, Inc.).

### Real-time quantitative polymerase chain reaction (qRT-PCR)

Following incubation with ZGDHu-1, TRIzol reagent (Takara, Dalian, China) was used to extract total RNA from cells according to the manufacturer's instructions. Reverse transcription to complementary DNA (cDNA) was achieved using a PrimeScript RT reagent kit with a gDNA eraser (Takara). Amplification reactions were performed using a SYBR Premix Ex Taq II kit (Takara) and a LightCycler 480 real-time PCR system (Roche Diagnostics, Mannheim, Germany). The Mcl-1, Bcl-2, Bcl-XL and Bax expression levels were determined in duplicate using predesigned Assay-On-Demand probes (Invitrogen, Beijing, China) on a LightCycler 480 real-time PCR system. Each gene's relative expression level was quantified using the comparative cycle threshold (Ct) method (ΔΔCt) and b-actin as the endogenous control. The expression level was given in arbitrary units, using control (untreated cells) or Jeko-1 cells as a reference.

### Statistical analysis

All data were collected from three independent experiments. Values, expressed as the means ± standard deviations (SD), were analyzed using Student's t test, and P values less than 0.05 were considered statistically significant.

## SUPPLEMENTARY MATERIALS FIGURES


