# Supplementary material for: ZGDHu-1 promotes apoptosis of mantle cell lymphoma cells
Source: Oncotarget. 2016 Dec 27;8(7):11659–75. doi: 10.18632/oncotarget.14274 (PMC5355294; doi:10.18632/oncotarget.14274)
Supplement: Supplementary file 1 [file oncotarget-08-11659-s001.pdf]

ZGDHu-1 promotes apoptosis of mantle cell lymphoma cells

SUPPLEMENTARY FIGURES

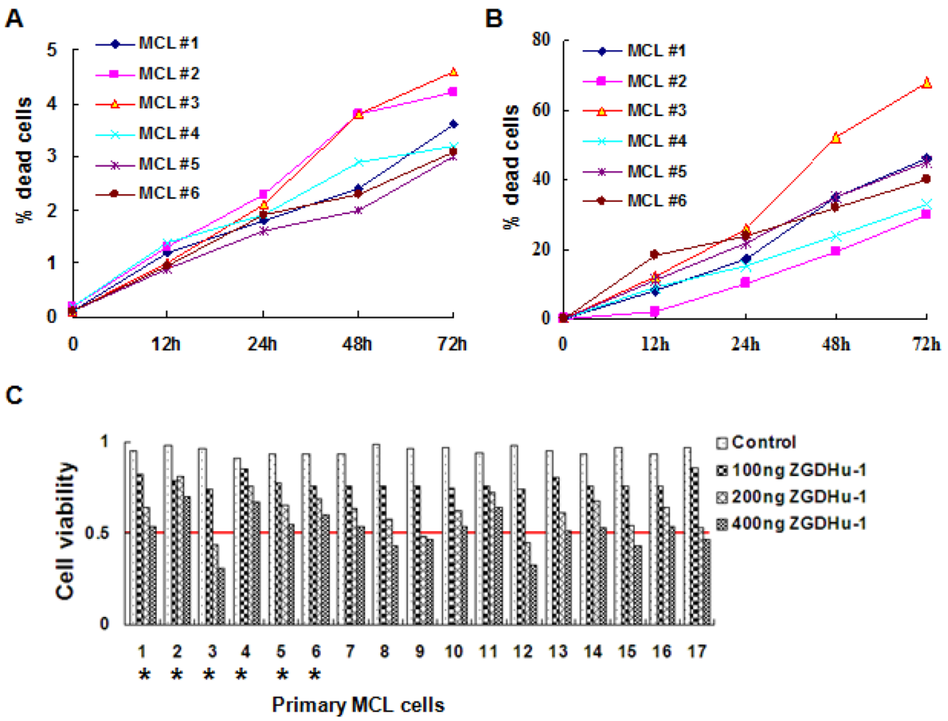

Supplementary Figure 1: Identification of ZGDHu-1 as a potent anti-lymphoma compound in primary MCL cells. A. Six primary MCL cells were cultured in 0.05% DMSO drug-free mediumfor 0 - 72 h. Cell viability was measured with PI staining and analyzed by flow cytometer. **B.** Six primary MCL cells were treated with 200 ng/ml ZGDHu-1 for 0 - 72 h, stained with PI and analyzed by flow cytometer. **C.** Seventeen primary MCL cells were treated with 100 - 400 ng/ml ZGDHu-1 for 48 h. Cell viability was measured using PI staining and flow cytometer.

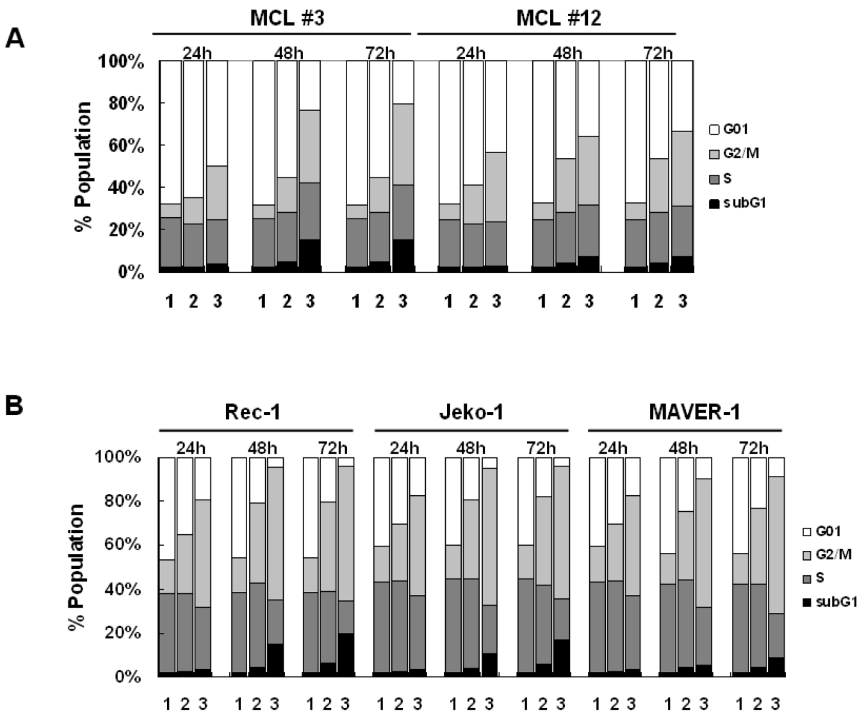

Supplementary Figure 2: ZGDHu-1 induces cell cycle G2/M phase arrest in MCL cells. A. Primary MCL cells from two MCL patients (MCL #3 and MCL #12) were cultured in 0.05% DMSO drug-free medium or 100 - 200 ng/ml ZGDHu-1 for 24 h, 48 h and 72 h. Quantifications of the proportions of cells in subG1, G01, S, and G2/M phases are listed for each experiment. 1. Control; 2. 100 ng/ml ZGDHu-1; 3. 200 ng/ml ZGDHu-1. **B.** Quantifications of the proportions of cells in subG1, G01, S, and G2/M phases are listed for three MCL cell lines cultured as above. 1. Control; 2. 100 ng/ml ZGDHu-1; 3. 200 ng/ml ZGDHu-1.

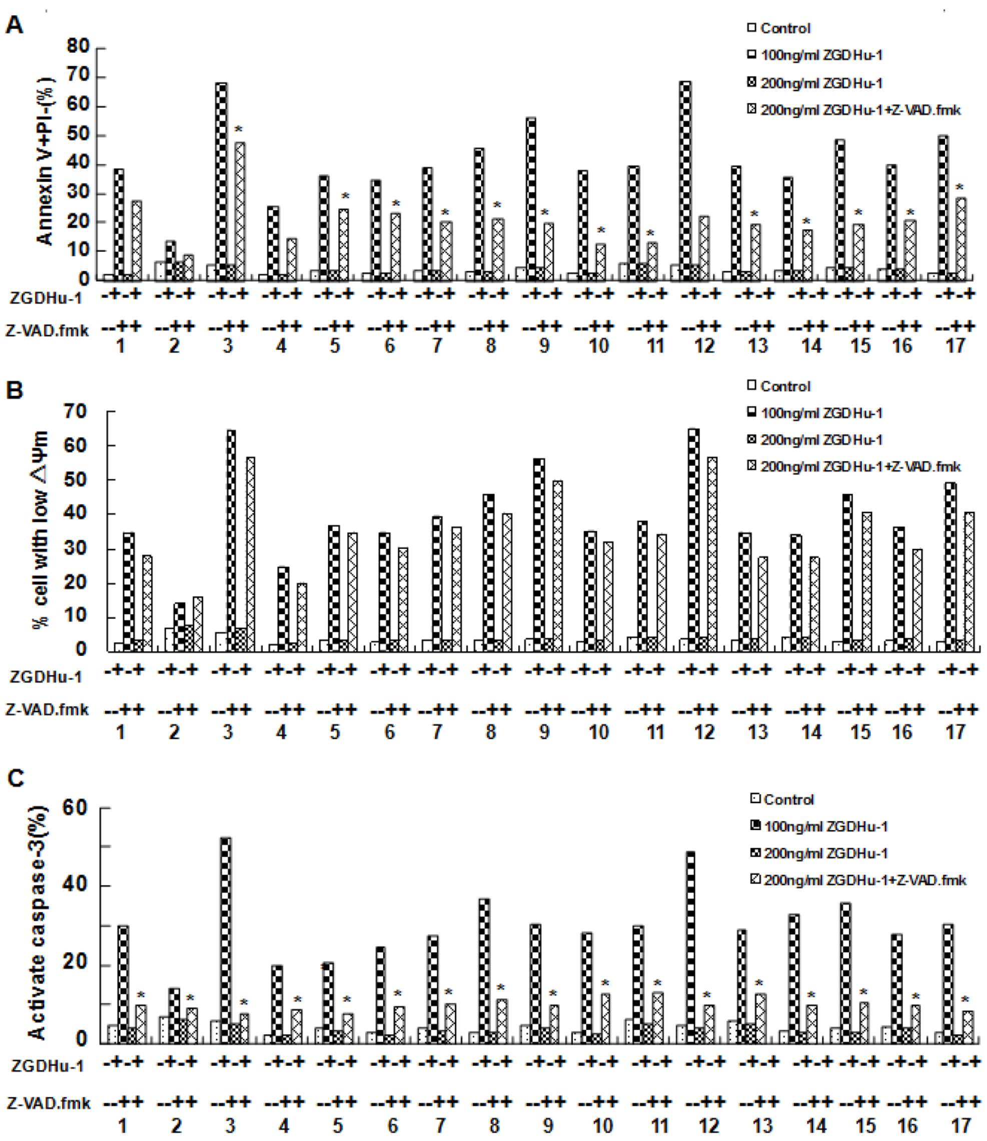

Supplementary Figure 3: ZGDHu-1 induces apoptosis in primary MCL cells. A. Seventeen primary MCL cells were treated with 200 ng/ml ZGDHu-1 in the absence or presence of Z-VAD.fmk for 48 h, stained with Annexin V/PI and analyzed by flow cytometry. * $P<0.05$ between ZGDHu-1 and GDHu-1+Z-VAD.fmk. **B.** ROS was analyzed by flow cytometry in 17 primary MCL cells following 200 ng/ml ZGDHu-1 treatment in the absence or presence of Z-VAD.fmk for 48 h. **C.** Activate caspase-3 was analyzed by flow cytometry in 17 primary MCL cells treated with 200 ng/ml ZGDHu-1 in the absence or presence of Z-VAD.fmk for 48 h. * $P<0.05$ between ZGDHu-1 and ZGDHu-1+Z-VAD.fmk.
